# A novel cognitive behavioural intervention with Theory of Mind (ToM) training for children with epilepsy: protocol for a case series feasibility study

**DOI:** 10.1186/s40814-019-0393-x

**Published:** 2019-01-19

**Authors:** Elizabeth Stewart, Cathy Catroppa, Suncica Lah

**Affiliations:** 1 School of Psychology, University of Sydney, 94 to 100 Mallet Street, Camperdown, Sydney, New South Wales 2040 Australia; 2 Australian Research Council Centre of Excellence for Cognition and its Disorders (ARC CCD), Sydney, Australia; 3 Murdoch Children’s Research Institute, Melbourne, Australia; 4 Royal Children’s Hospital, Melbourne, Australia; 5 University of Melbourne, Melbourne, Australia; 6 Australian Catholic University, Sydney, Australia

**Keywords:** Intervention, Treatment, Epilepsy, Theory of Mind, Social competence

## Abstract

**Background:**

Children with epilepsy have significant social impairments, yet evidence-based interventions to address these social difficulties are lacking. Emerging research has shown that social difficulties in children with epilepsy relate to underlying impairments in Theory of Mind (ToM). This paper outlines the protocol for a pilot study that will evaluate the feasibility, acceptability, and efficacy of a novel cognitive behavioural intervention with ToM training for children with epilepsy.

**Methods:**

The intervention will be evaluated in a single-arm case series feasibility study. Ten to 12 children with common forms of epilepsy (8 to 12 years old) will be recruited to participate in 4 small group workshops, held over 4 consecutive weeks. Parents will attend a brief review at the end of each session with their child. Children will complete 4 one-to-one assessments with an investigator assessing ToM and social competence: twice at baseline (4 weeks and 1 day before the intervention), at post-intervention (last day of the intervention) and at follow-up (4 weeks post intervention). Parents will complete online questionnaires at these same 4 time points assessing ToM and social competence of their child. Parents and children will both complete a weekly measure of social competence from baseline 1 to follow-up. Following completion of the intervention, parents will complete two standardised questionnaires assessing treatment acceptability and barriers and facilitators to attendance; children will complete a single questionnaire on treatment acceptability. Information about feasibility outcomes (i.e. recruitment and retention, processing time, suitability of tasks) will be gathered by investigators during the trial. Together, outcomes will be used to refine research methods and make a decision about whether the intervention should be evaluated in a larger scale trial.

**Discussion:**

To our knowledge, this is the first psychosocial intervention to address social competence problems in children with epilepsy. Findings will provide information about a potentially effective treatment that could improve longer term social outcomes for this group.

**Trial registration:**

Australia and New Zealand Clinical Trials Register (ANZCTR): ACTRN12618000974202, registered June 8 2018.

**Electronic supplementary material:**

The online version of this article (10.1186/s40814-019-0393-x) contains supplementary material, which is available to authorized users.

## Background

Epilepsy is a common neurological condition in childhood that is associated with significant psychosocial difficulties: reduced social competence, social isolation and high levels of psychological distress [1, 2]. At present, there are no effective, evidence-based interventions to treat social difficulties in children with epilepsy. This is concerning as social difficulties that emerge in childhood tend to persist in adults with epilepsy and have a negative impact on patients’ social and psychological adjustment and quality of life [3, 4]. Early intervention may reduce the social and emotional burden of epilepsy; however, researchers have struggled to develop effective psychosocial treatments, in part due to limited understanding of the neurocognitive underpinnings of social difficulties in this group. Promisingly, emerging evidence from empirical studies has shown that children with common forms of epilepsy, including genetic generalised epilepsy (GGE) and temporal lobe epilepsy (TLE) have significant impairments in Theory of Mind (ToM), which are related to social competence and social communication problems [5–7]. This has led to suggestion that training children’s ToM may be an effective way of improving social outcomes in this group [8, 9].

Theory of Mind (ToM) refers to the ability to understand the thoughts, intentions, beliefs and emotions of others and oneself [10]. ToM is comprised of component skills including the capacity to understand mental states, including beliefs and knowledge (cognitive ToM), and to identify and communicate emotions (affective ToM) [10]. Interventions targeting ToM have been developed and trialled for children with other neurodevelopmental conditions, including autism spectrum disorder (ASD) and hearing impairment [11–13]. For these groups, ToM interventions have been broadly effective at improving ToM [14, 15], with a recent meta-analysis finding medium-size improvements in children’s ToM following training (Hedges’ *g* = 0.747) [14]. Improvements in everyday social competence following ToM training have been documented in some studies [16, 17] but not others [11, 12]. The efficacy of prior interventions was found to depend on whether the components included in training were appropriate for age and ability level of the target group [14]. This included the suitability of ToM modules (i.e. targeting cognitive and/or affective ToM) and whether strategies were included to assist with the acquisition and/or generalisation of ToM skills (i.e. language exercises, executive functioning [EF] strategies and cognitive behavioural therapy [CBT] skills).

Interventions targeting early developing cognitive ToM (i.e. false belief understanding), which matures between 3 to 5 years, were found to produce large improvements in ToM in pre-schoolers [18, 19] but had little effect on ToM in children over 8 years old [20]. For this age group (≥ 8 years old), interventions that targeted advanced cognitive and/or affective ToM, which emerge between 8 to 12 years old, were successful at achieving improvements in ToM [21]. This is unsurprising, as older children are unlikely to benefit from training in skills that they have already acquired at an earlier developmental age. In addition, several studies included strategies to target cognitive skills that develop alongside ToM, such as language [16, 21] and EF [17, 20]. Including these strategies was found to be beneficial for children with impaired language and EF (e.g. children with ASD) who showed improvements in ToM when these components were included, but not when they were omitted from training [11, 20]. Finally, a few studies included CBT skills alongside ToM training and these studies also reported improvements in children’s everyday social competence [16, 17]. This is consistent with research suggesting that understanding the relationship between thoughts, feelings and behaviours is critical for applying ToM reasoning to the social world [22]. Finally, contextual factors relating to the structure, timing and frequency of sessions have also been found to impact treatment outcomes. Interventions with longer individual sessions and shorter overall training periods were associated with larger improvements in ToM in a recent meta-analytic review [14]. Additionally, involvement of same-age peers, small group sessions, active involvement of group facilitators and inclusion of parent-review sessions have been found to enhance engagement and learning among children in prior interventions targeting ToM and broader social skills [14, 23].

It is unclear whether interventions developed for other clinical groups would be appropriate and/or beneficial for children with epilepsy. This is because children with epilepsy have a unique cognitive and behavioural profile, including impairments in general language abilities and EF that often occur in the absence of impaired intellectual quotient (IQ) [24, 25] and impairments in social competence, social communication and social cognition in the absence of additional features of ASD (e.g. autistic styles of communication and behaviour, such as idiosyncratic language) [26]. Given that the success of prior ToM interventions has depended upon the suitability of treatment components for the target group, strategies required for children with epilepsy may differ from other clinical groups. Thus, we sought to develop a tailored intervention specifically for children with epilepsy, aged 8 to 12 years old. We selected this age range in middle childhood as it is a time of rapid growth in ToM when social and emotional difficulties in patients with epilepsy often emerge [8]. We developed the intervention in accordance with the Medical Research Council (MRC) guidelines for intervention development [27] to address shortfalls of prior ToM interventions in a number of ways. First, the intervention targets advanced cognitive and affective ToM, which have been found to be impaired in children with epilepsy who are between 8 to 12 years old [5–7]. Second, the intervention includes strategies to assist with language and EF, which are commonly impaired in young epilepsy patients [25, 28, 29]. We have included both mental-state [21] and emotion vocabulary [16] language activities, similar to those included in prior ToM interventions targeting the same age group, as research suggests that these are important for developing cognitive and affective ToM, respectively [14]. Third, the intervention includes basic CBT skills alongside ToM training, as this is regarded as important for translating ToM reasoning into observable social behaviours [22]. Finally, we have included various contextual factors that have been associated with better engagement and larger improvements in ToM and social skills (e.g. involvement of parents and same-age peers, small group sessions) [14, 23].

To our knowledge, this is the first intervention that has been designed and trialled specifically for children with epilepsy to target ToM and social impairments. Given the preliminary nature of the intervention, pilot testing is crucial to ensure that it is feasible, acceptable and safe prior to implementation in a larger scale trial. The protocol has been developed and is described in accordance with the Standard Protocol Items: Recommendations for Interventional Trials (SPIRIT) guidelines (see Additional file 1) [30]. The intervention is described according to the Template for Intervention Description and Replication (TIDieR) checklist (see Additional file 2) [31].

### Objectives

The primary objectives of the study are to assess feasibility and acceptability of the intervention for children with epilepsy (8 to 12 years old) with respect to the following factors:

1. Recruitment and retention rates (including attendance at sessions).
2. Processing time for intervention modules.
3. Suitability of tasks (including ability and willingness of children to participate in activities), as determined by observations made by investigators during sessions.
4. Overall acceptability of the intervention as a comprehensive programme, as determined by responses from parents and children on standardised questionnaires.
5. Barriers and facilitators to attending the programme, as indicated by parents of children who participate on a standardised questionnaire.

The secondary objective is to determine whether the intervention is effective at improving

(1) ToM and (2) social competence, as determined by children’s performance on behavioural tasks as well as responses from children and parents on standardised questionnaires.

### Trial design

Pilot and feasibility study guidelines were consulted in designing and planning the study [32, 33]. The study will employ a double baseline case series design, and the intervention will be run as a single-arm open-label pilot. The framework for the study is exploratory. The decision to conduct an open-label pilot was guided by research by Leon and Davis [33], who state that open-label studies in which participants are recruited to known albeit experimental interventions with no risk of receiving placebo are best practise in pilot trials. A case series was selected over a single-case or larger scale randomised controlled trial (RCT) for feasibility, ethical and methodological reasons. Case series are increasingly used in cognitive neuropsychology and are considered more robust and generalisable than single-case designs [34, 35]. Case series involve recruiting a small sample of children to a single experimental condition and assessing individual change in performance across the course of treatment [35]. The most robust and validated method for assessing within-subject change in case series is visual analysis, a method that requires each participant to be assessed at least twice prior to starting treatment (i.e. double baseline). A double baseline approach will be achieved in the current trial by assessing children twice before treatment begins (i.e. baseline 1 and 2). This will provide a stable measure of performance before the intervention, which can be compared to performance on the same measures at post-intervention and follow-up. This approach is common in case series interventions and allows researchers to conclude with some certainty that improvements are attributable to a given treatment and not to practice effects or the passage of time [35]. A double baseline design is also required for the approach that we will use to evaluate outcomes (i.e. visual analysis, see the “Statistical methods” section for more details about this method). A larger scale RCT was not considered appropriate for the current study as this is the first time the intervention will be trialled, and it does not have known positive effects.

## Methods

### Study setting

The study will be conducted by researchers and clinicians at The University of Sydney, the Sydney Children’s Hospital at Randwick and the Children’s Hospital at Westmead.

### Participants and sample size

Ten to 12 children with epilepsy (TLE and/or GGE, aged 8 to 12 years old) will be recruited for the study. Children with TLE and GGE have been selected as ToM impairments and associated social problems have been documented in these patient groups [5–7]. ToM has not yet been studied or not been found to relate to social impairments in children with other forms of epilepsy [36]. Moreover, in a recent systematic review and meta-analysis, adults with parietal and occipital lobe epilepsy were not impaired in ToM relative to controls calling into question whether children with these conditions would have ToM deficits [36]. The sample size has been selected based on guidelines provided by Julious [37] and Whitehead [38] for conducting pilot trials. Another consideration in selecting sample size was that smaller groups have been found to be more effective and engaging than larger groups in prior ToM and social skill interventions [14, 23]. Although the intended sample size is small, the primary outcomes will be able to be addressed if just two children complete the programme, as case series require a minimum sample size of two [35]. By inviting 10 to 12 children to participate, we will start with a sample size that is 5 to 6 times greater than required for dissemination of results.

#### Eligibility criteria for children with epilepsy

Children will be considered eligible if they meet the following criteria: (i) a current diagnosis of GGE or TLE, confirmed by treating neurologists, (ii) aged between 8 to 12 years old, (iii) fluency in the English language, (iv) assessed to have a full scale intellectual quotient (FSIQ) > 70, (v) absence of major developmental/psychiatric conditions (i.e. autism), sensory/motor impairments (i.e. cerebral palsy, significant visual or hearing impairments), major neurological disorder (e.g. severe brain injury), systemic and metabolic disorders that could lead to cognitive impairment, (vi) no clinical signs of drug intoxication/drug and alcohol abuse, (vii) absence of a neurological procedure/surgery in the past 6 months, and (viii) currently controlled seizures with medication, meaning that the child has experienced a stable seizure frequency for the past 6 months, as assessed by treating neurologists at enrolment. There is no seizure frequency cutoff. Children will be excluded if they (i) are undergoing active AED changes, as this could affect seizure frequency or other aspects of the child’s functioning (e.g. reduced concentration, fatigue) and may limit their ability to engage in therapy, or (ii) are currently engaged in another form of treatment for social difficulties (individual or group therapy), as concurrent treatment may add a source of bias to results. Children who have previously attended psychology sessions, but are not currently engaged in treatment, will not be excluded and will be considered eligible for the study. All children will be instructed to continue with routine clinical care for their epilepsy during the study.

#### Eligibility criteria for investigators

Investigators from The University of Sydney who will conduct intervention and assessment sessions are psychologists and neuropsychologists who hold full registration with the Psychology Board of Australia. Investigators from participating hospitals are neurologists who will be responsible for confirming diagnosis of epilepsy, current seizure stability and completing a measure of overall epilepsy severity for each participant enrolled in the trial. All investigators will be registered with Australian Health Practitioners Registration Agency (AHRPA) in their respective professions.

### Intervention

A brief overview of the intervention is provided in Table 1. The intervention consists of two phases: an introductory CBT phase (week 1, modules 1 to 3) and an active ToM training phase (weeks 2 to 4, modules 4 to 12). In the introductory CBT phase, children will learn about the relationship between thoughts, feelings and behaviours and complete activities to show that two people can think and feel differently in the same situation and subsequently respond in different ways. They will also complete language exercises focusing on mental state and emotional vocabulary. In the active training phase, children will be introduced to the concept of ToM through three roles/positions: the thinker (understands another person’s thoughts, beliefs and intentions [cognitive ToM]), the feeler (understands another person’s emotional state [affective ToM]) and the responder (can use ToM knowledge to generate an appropriate social response). Strategies to help children take each position will be summarised on self-instructional prompt cards, which will act as a strategy to assist with EF. Children will then be introduced to eight social scenarios that require ToM, in which characters have differing perspectives, beliefs, knowledge or emotional reactions to a situation/event: white lies, misunderstandings, forgetting, irony, pretending, figures of speech, persuasion and double bluff. These scenarios have been selected from the strange stories task, an advanced test of cognitive and affective ToM, for two reasons: (i) children with epilepsy have significant difficulties on this task [5–7] and (ii) a prior ToM intervention found that utilising these specific social scenarios was beneficial for training ToM in children aged 8 to 12 years old [21]. Each subsequent module will focus on one scenario and children will practise applying ToM knowledge and CBT skills to respond. Investigators will provide feedback and prompting to help children modify their performance and generate appropriate responses. Activities will be taught via a variety of mediums including written social stories, videos, role-plays, group discussions and paper and pen tasks. Parents will join children for the final 15 to 30 min of each session, during which a summary of skills will be provided and homework will be allocated. Homework will extend upon the content of intervention sessions. All activities are collated within intervention workbooks, which will be distributed to children and parents on the first day of training. All materials can be accessed by contacting the lead author of this paper.

**Table 1:** Brief overview of the intervention

Session^a^	Module	Content
Phase 1. Introductory cognitive behavioural therapy (CBT) phase		
1	1	Thoughts
	2	Feelings
	3	Thoughts, feelings and behaviours
Phase 2. Active Theory of Mind (ToM) training phase		
2	4	Theory of Mind
	5	White lies
	6	Misunderstandings
3	7	Forgetting
	8	Irony
	9	Pretending
	10	Figures of speech
4	11	Persuasion
	12	Double bluff
	13	Review

^a^Session division is a plan only. Content of modules is subject to change based on outcomes of the pilot trial

### Discontinuing/modifying the intervention for individual participants

The intervention has been designed as a comprehensive programme, meaning all children are expected to be able to complete all modules regardless of their baseline cognitive skills (i.e. regardless of whether children are assessed to have impaired language or EF). Nevertheless, investigators will monitor the performance of each child and modify activities, as best as they can, to meet the individual needs of the child during sessions. This individual tailoring is within the scope of investigators’ training and experience as child psychologists and neuropsychologists; in spite of the group setting, we do not anticipate difficulties achieving this individual tailoring, as the group size will be small and multiple investigators will be present for each session. In addition, the intervention may need to be modified or discontinued if (1) an adverse event occurs, including events that are causally related to the intervention, not related or events that have an unidentified cause. Consultation and advice from the governing human research ethics committee (HREC) and (if necessary) from clinicians from participating hospitals and The University of Sydney who are external to the project will be used to determine the most appropriate course of action, or (2) withdrawal of participant consent. Participants will be informed at enrolment and throughout the study that they are free to withdraw at any time without affecting their current or future relationship with treating clinicians, participating hospitals or The University of Sydney. A withdrawal of consent form will be provided with the participant information and consent form (PICF) at the start of the study and will be given to participants at their request (see Additional file 3).

### Fidelity/adherence during the intervention

Homework tasks are optional and no compulsory activities are required of children and parents outside of sessions. Nevertheless, adherence to homework will be monitored by asking parents at the start of each session whether they completed homework activities and recording this information for each child, each week. Although monitoring adherence/fidelity is important, particularly when assessing whether adherence to certain aspects of treatment affects outcomes in a trial, the primary objective of this study is to assess feasibility of the intervention. We are primarily interested in whether homework is perceived to be beneficial, achievable and relevant for families. Failure to complete homework or feedback that activities are irrelevant or too time-consuming will be used along with other information as a measure of treatment acceptability.

### Outcomes

**Fig. 1:**
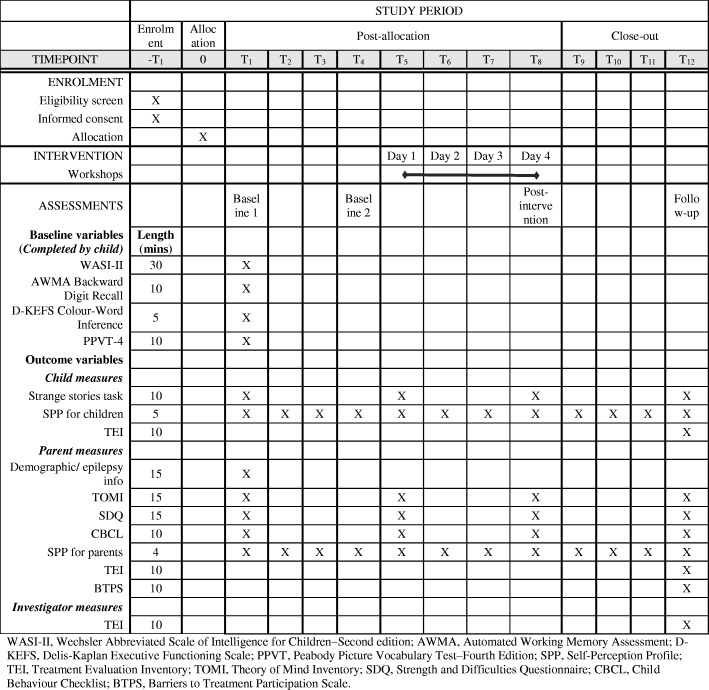
Summary of enrolment, intervention and assessment procedures based on the SPIRIT figure [30]

Figure 1 provides a summary of standardised outcome measures to be completed during the study using the SPIRIT template.

#### Primary outcome measures

The primary outcomes relate to feasibility and acceptability of the intervention and will be evaluated in the following ways:

1. Recruitment and retention rates. Recruitment will be deemed feasible if it is possible to recruit 40 to 50% of children who are invited to participate. A retention rate of 80% will be considered acceptable (i.e. if 10 children commence the study, at least 8 should complete the intervention). Completion of the intervention is defined as attending at least 75% of sessions (i.e. 3 of 4 days, which must include the first day of training). These rates are based on findings from two recent reviews of recruitment and retention in pilot trials in which recruitment rates (i.e. participants identified as eligible who were enrolled and randomised) ranged from 42.0 to 50.4%, and retention rates ranged from 78.9 to 82.3% (i.e. attrition of 21.1 and 17.7%, respectively) [39, 40].
2. Processing times for intervention modules. Investigators will record the length of time it takes to complete each module during intervention sessions. Modules lasting < 20 min or > 50 min will be evaluated and modified. This timeframe has been selected as it is in keeping with standard psychology sessions and ensures adequate content can be covered without extending children beyond their attentional capacity.
3. Suitability of tasks (including ability and willingness of children to participate in activities). Investigators running the intervention will observe children’s participation during sessions and record whether the child participated actively (i.e. completed task and provided at least one verbal or written response), participated passively (i.e. completed task but did not provide verbal or written responses) or refused to participate. Moreover, questions that elicit no response or an unexpected response indicating misunderstanding of concepts will be recorded for review and modification. Based on this, each module will be classified as suitable without modification (retain as is), suitable with modification (amend and modify) or unsuitable (discard or replace).
4. Overall acceptability of the intervention will be evaluated with the Treatment Evaluation Inventory (TEI) [41], which will be administered to parents and children upon completion of the intervention. The TEI consists of 19 items that ask respondents to rate how acceptable, suitable and achievable a given treatment is on a 7-point Likert scale (1 = not at all to 7 = very much). The TEI generates a total score (range = 19 to 133) and two subscale scores: child progress (range = 11 to 77) and acceptability of treatment (range = 8 to 56). Investigators running the intervention will complete a parallel version of the TEI for each child. The TEI therapist version contains 15 items that are scored on the same 7-point Likert scale and generates a total score (range = 13 to 87) and two subscale scores: child progress (range = 6 to 24) and child improvement (range = 9 to 63). Raw scores are summed to make up each subscale, and total scores are converted into percentage total scores (range = 0 to 100). Higher scores indicate greater progress, acceptability or improvement. A total percentage score ≥ 80 on each subscale of the TEI is considered adequate acceptability for novel treatments [42]. Failure to meet this minimum rating will be taken into consideration, along with other outcome measures, in assessing the intervention. Scores that differ between parents, children and investigators on the TEI will be followed up in debriefing interviews to determine possible causes of discrepancy. The TEI was selected as it has been found to be a valid and reliable measure of treatment acceptability that contains parallel scales for children, parents and therapists to complete [43]. Semi-structured interviews with parents will be conducted upon completion of the intervention to obtain additional, qualitative information about treatment acceptability and clarify any findings from questionnaires.
5. Barriers and facilitators to attendance will be evaluated with the Barriers to Treatment Participation Scale (BTPS) [44], which will be completed by parents at the end of treatment. The BTPS is a 44-item questionnaire that asks respondents about potential barriers to completing treatment on a 5-point Likert scale (1 = never a problem to 5 = very often a problem). The scale yields a total score reflecting perceived barriers to participation in treatment and four subscale scores: stressors and obstacles that compete with treatment (e.g. responsibilities for other children, time constraints, family conflict), treatment demands and issues (e.g. treatment was confusing, too long, costly, difficult or demanding), perceived relevance of treatment (e.g. treatment was seen as relevant to the child’s problems, was viewed as important and met with parent expectations) and relationship with the therapist (e.g. parent’s alliance and bonding with the therapist including liking of and perceived support from the therapist). The BTPS was chosen as it covers the major barriers reported by parents [45] and young people [46] to accessing psychosocial treatments in two recent systematic reviews. Frequency data will be inspected to determine which items are most commonly endorsed on this measure by parents.

#### Secondary outcome measures

##### Theory of Mind (ToM)

The strange stories task [47] will be used as the primary measure of change in ToM. Parallel versions of the task will be administered to children at baseline 1, baseline 2, post-intervention and follow-up. The strange stories task contains 12 social stories in which there is a discrepancy in characters’ perspectives or beliefs and children must explain the discrepancy with reference to characters’ thoughts and feelings. Responses are scored on a 3-point Likert scale (0 = no reference to thoughts and feelings, 1 = reference to thoughts or feelings, 2 = reference to thoughts and feelings). A single total ToM score is generated by summing items (range = 0 to 24). Three control stories from the strange stories task that are comparable in length and difficulty, but contain no reference to thoughts or feelings of characters, will also be administered to obtain a measure of general story comprehension (range = 0 to 6). The strange stories task was selected as empirical studies have found that children with GGE and TLE have significant impairments on this task [6, 7]. An additional (secondary) measure of change in ToM will be obtained with the Theory of Mind Inventory (TOMI) [48], which will be completed by parents at the same 4 time points. The TOMI is a 42-item parent-report questionnaire that assesses ToM in children aged 3 to 17 years old. Parents rate how accurate statements are for their child on a 5-point Likert scale (1 = definitely not true to 5 = definitely true), and scores are summed to generate a total raw score (range = 42 to 220), which is converted into a percentage total score (range = 0 to 100). The TOMI is being included as it has been found to be sensitive to change following interventions in other clinical populations [48].

##### Social competence

The social competence subscale of the Self-Perception Profile (SPP) [49] will be used as the primary measure of change in social competence. The SPP will be completed weekly by both children and parents from baseline 1 to follow-up. The SPP consists of parallel child (6 items) and parent (3 items) forms, which assess a child’s social skills, peer acceptance and popularity among peers. For each item, the rater chooses one of two statements that best describes the child and then rates how true that statement was over the past week (really true or sort of true). Each item is scored from 1 to 4, with a total score calculated by averaging all items (range = 1 to 4). A higher score indicates better social competence. The SPP has been selected as it is considered a valid and reliable self-rated measure of social competence in children, is sensitive to change following interventions and includes a parent-rated version that parallels the child scale, which is considered critical for adequately assessing social outcomes [50]. Additional (secondary) measures of social competence will be obtained with standardised, parent-report questionnaires: the Strength and Difficulties Questionnaire (SDQ, total problems subscale; total raw score will be used for interpretation) [51] and the Child Behavior Checklist (CBCL, social competence subscale; *t* scores will be used for interpretation) [52]. These will be completed at baseline 1, baseline 2, post-intervention and follow-up. They are being included as (i) impairments on these measures were related to impairments in ToM in prior studies of children with epilepsy [6, 7] and (ii) these scales are widely employed and validated questionnaires, which may be useful for future trials and clinical care. Ratings that differ between parents and children will be followed up in debriefing interviews to determine possible causes of discrepancy. Contradictory findings on different measures will be used to revise research methods and select outcome measures for an ongoing trial.

#### Baseline cognitive measures

Figure 1 outlines cognitive tests to be completed by children assessing IQ, EF and general language skills. These will be completed at baseline 1 only. The Wechsler Abbreviated Scale of Intelligence for Children–Second edition (WASI-II; Vocabulary, Similarities, Block design, Matrix Reasoning) [53] will be used to obtain a brief estimate of general intellectual abilities. The Peabody Picture Vocabulary Test–Fourth Edition (PPVT–4) [54] will be used as a measure of receptive vocabulary (general language skills). The Automated Working Memory Assessment (AWMA) Backward Digit Recall subtest [55] will be used to assess verbal working memory. The Delis-Kaplan Executive Functions System (D-KEFS) Colour-Word Reading test [56] will be used as a measure of behavioural inhibition. These are being included as knowledge of children’s baseline cognitive skills may provide useful information about individual participants ability to engage with or benefit from activities, which may be useful for drawing conclusions about the suitability of tasks using the case series approach [35].

#### Demographic and clinical variables

Semi-structured interviews with parents at baseline 1 will be used to obtain information about demographic and epilepsy factors (i.e. age of seizure onset, seizure frequency, current medications, surgical status), the child’s current social and emotional functioning and any psychosocial treatment they have received in the past. Treating neurologists will complete the Global Assessment of Severity of Epilepsy (GASE) [57] at baseline 1 to obtain an overall measure of epilepsy severity. The GASE is a validated, single-item questionnaire that asks clinicians to rate severity of epilepsy from 1 to 7, with a higher score indicating more severe epilepsy.

### Participant timeline

**Fig. 2:**
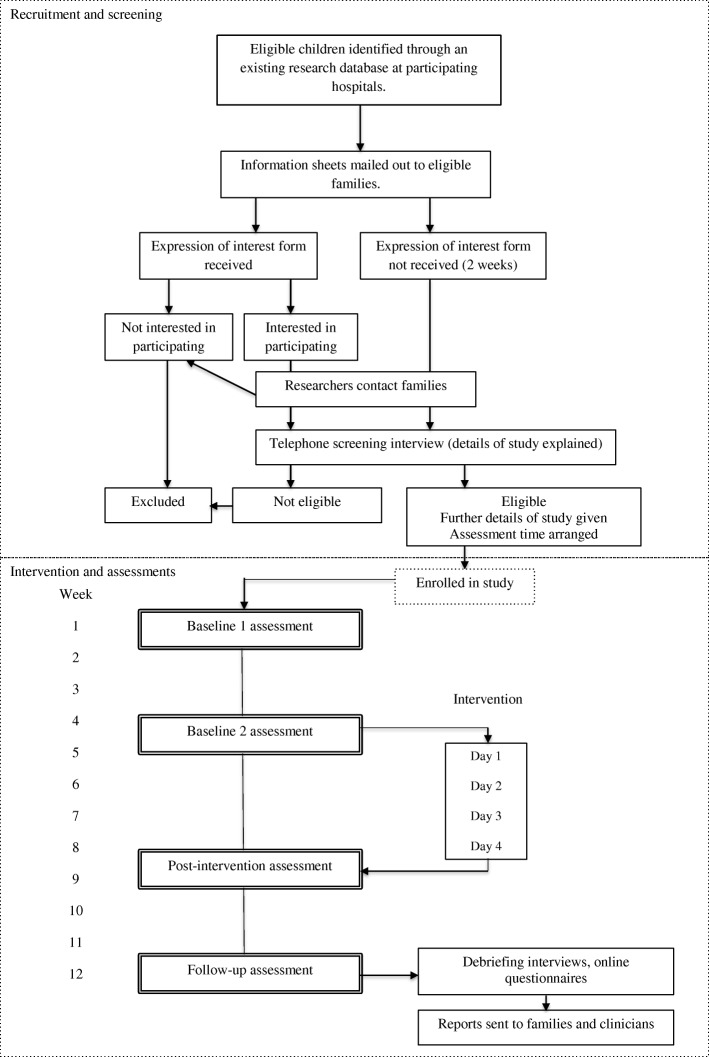
Study design diagram: procedures for recruitment and delivery of the intervention and assessments

Figure 2 provides an overview of procedures for recruitment, delivery of the intervention and assessments.

#### Procedure for recruitment

Children with epilepsy will be recruited from an existing research database at participating hospitals. All children invited for the current study participated in a previous cross-sectional study conducted between 2015 and 2017, and parents/guardians have consented to be re-contacted for future studies. In the previous study, children completed tasks at a single time point assessing ToM and relevant cognitive skills (e.g. IQ, EF). Parents completed questionnaires assessing social competence of their child. Parents and children received a brief rationale for why we were studying social cognition in epilepsy, but no discussion or explanation was provided for an intervention nor was this available at the time that the cross-sectional study was conducted. Participants identified as eligible will be mailed out PICFs, which contain an expression of interest form for parents to sign and return to investigators. As we are only recruiting 10 to 12 children, the first eligible children in the database will be re-contacted. Participants who express interest will be contacted by telephone and a screening interview will be conducted to confirm eligibility. Participants who do not return the expression of interest form within 2 weeks of it being mailed out will be contacted by telephone to explain the study and determine whether they are interested and eligible. If the parent and child are eligible and interested, parents will be provided with details of the study (i.e. dates and locations of sessions) and a time will be arranged for the initial assessment session.

#### Procedure for intervention delivery and assessments

Children will participate in 4 small group workshops held over 4 consecutive weeks at the Psychology Clinic at The University of Sydney. Each workshop will last for approximately 2 to 3 h and will be divided into 2 to 3 sessions (20 to 50-min each), each separated by a 10- to 15-min break. The final 15- to 30-min of each session will be a review with parents and children.

Children will complete 4 one-to-one assessments with an investigator at baseline 1 (4 weeks before the intervention begins), baseline 2 (1 day before the intervention begins), post-intervention (last day of intervention) and follow-up (4 weeks after the intervention). During assessments, children will complete a behavioural task assessing ToM and parents/guardians will complete questionnaires that assess ToM and social competence of their child. A brief weekly measure of social competence will be completed by both parents and children from baseline 1 to follow-up. Assessments with children will be conducted individually, face to face, at The University of Sydney Psychology Clinic or participants homes. Parent questionnaires and weekly measures will be completed online.

Following completion of the intervention, parents and children will attend face-to-face debriefing interviews to discuss the child’s participation and provide feedback. Parents will complete two online questionnaires assessing treatment acceptability and barriers and facilitators to attending the programme. Children will complete a single questionnaire assessing treatment acceptability that parallels the questionnaire completed by parents. These questionnaires will be completed before the debriefing interview to minimise bias in responding based on clinician feedback.

### Data collection and data management

The intervention and assessments will be conducted by psychologists and neuropsychologists who are trained and experienced in administering the tasks included in the assessment battery and intervention sessions. Thus, no additional training will be required for any of the investigators. To promote participant retention and completion of follow-up, home visits will be offered for assessments, as this has been found to almost double retention rates in pilot studies [58]. Home visits will not be offered for intervention sessions, as being exposed to treatment in a different learning environment may add a source of bias to results. Participants who discontinue or withdraw prior to completion of the study will not be required to complete any further assessments, as per requirements of the governing ethics committee. It is not anticipated that participants could deviate from the study protocol in any way other than discontinuing sessions.

Each participant will have a unique identification (ID) code assigned to them at the start of the study, which will be used to de-identify data and link information collected on paper and in electronic forms. During the study, de-identified data will be stored securely in the original paper and electronic copies in locked filing cabinets and password-protected files, respectively, at The University of Sydney. Codes and consent forms will be stored separately to the files themselves in a separate locked filing cabinet. After completion of the study, all data will be converted to secure electronic files and stored in password-protected files at The University of Sydney. Data will be retained until participants are 25 years of age or for a minimum of 15 years (whichever is later) as per the Australian Code for the Responsible Conduct of Research [59]. Data will only be accessible to study investigators listed on the ethics application. There are no external sponsors or other involved parties that will have access to data.

### Statistical methods

Primary analyses will report results relating to feasibility outcomes, which do not rely on quantitative statistical tests. Secondary analyses will involve data pertaining to children’s performance on tasks of ToM and social competence. Individual participant scores on secondary outcome measures will be plotted graphically and visual analysis will be used to assess change in performance from baseline 1 to follow-up. Visual analysis involves inspecting performance during an intervention and at follow-up and comparing this to a baseline mean (i.e. average of scores at baselines 1 and 2) [60]. Although new quantitative methods have been devised to estimate effect sizes in case studies, the use of these measures has been advised against in pilot studies due to the potential for inaccurate interpretation of results with unpowered samples [61]. Thus, visual analysis along with inspection of simple, descriptive statistics was considered the most appropriate method for the current study. Both primary and secondary analyses will be used to draw a conclusion about whether the study should be expanded into a larger scale trial. Given the small sample size and lack of quantitative statistical tests, we do not anticipate that statistical methods will be required to handle missing data; however, missing data for individual participants will be reported in papers arising from the research with an explanation of why data was not obtained.

### Overall success of the intervention

The overall success of the intervention and decision to evaluate it in a larger scale trial will be determined with both primary and secondary outcomes. A final decision about the protocol will be made upon completion of the study [32]:

- Stop (main study not feasible). None of the primary feasibility outcomes are met *and* a negative trend is observed in secondary outcomes *or* other adverse effects are reported.
- Continue but modify protocol (feasible with modifications). One or more of the primary feasibility outcome measures are not met.
- Continue without modifications (feasible as is). All of the primary feasibility outcomes are met *and* a positive trend is observed on both secondary outcomes (ToM and social competence).

Trends on secondary outcomes will be determined with visual analysis, as described in the “Statistical methods” section. A negative trend would be indicated by scores that fall below the baseline mean and positive trends by scores that fall above the baseline mean [60]. Scores that are comparable to baseline or fluctuate around the baseline mean would indicate neither a positive nor negative trend. A negative trend for ≥ 50% of participants would be sufficient to stop the main study (decision a). A positive trend on ≥ 50% of participants will be considered sufficient to continue the study, with or without modifications (decisions b or c). Smith [60] provides further details about this method. Participants included in the pilot study will not be included in an ongoing trial that results from this research to reduce the potential for bias in results.

### Monitoring and harms

A formal data monitoring committee (DMC) was not considered necessary for the current study given its short duration and minimal anticipated risks to participants [30]. Similarly, an auditing procedure is not in place nor was this required by the governing HREC given the short duration of the intervention, single-centre administration and minimal number of anticipated recruits. A formal DMC and auditing procedure will be established if the research progresses to a larger scale trial. Children’s progress will be monitored during the study by investigators running the intervention to ensure safety and reduce risk of harm. There are no significant foreseeable risks to children participating in this study; all tasks included in the intervention and assessments are safe and similar to activities that are routinely used in child therapy and neuropsychological assessments. Nevertheless, if children experience distress or wish to stop participating at any point, investigators will deal with this appropriately. Adverse events will be immediately reported to the governing HREC and dealt with appropriately at the time they are reported. Investigators will arrange for appropriate psychological support, which may include external referral if deemed appropriate by the governing HREC.

### Consent and confidentiality

Informed consent will be obtained from all participants prior to the first assessment by study investigators. Parents/guardians and children will be provided with written PICFs that explain the study in clear, age appropriate language (see Additional file 3 for PICFs that will be used for the study). Written consent will be obtained from parents/legal guardians. Children who are deemed by investigators to have the requisite capacity and maturity to understand the research will also be asked to provide written consent by countersigning the parent/guardian consent form. As per the child consent guidelines developed by the Australian Paediatric Research Ethics & Governance Network (APREG) [62], children who are not considered to have the required capacity/maturity to consent will not sign the consent form and the signature of their parent/guardian will be considered sufficient, provided the child has been involved in the discussion and their decision to participate or not has been respected, which is also consistent with the policy of the governing HREC. Participant confidentiality will be maintained by collecting and storing de-identified data and disseminating findings in a way that ensures participants are not individually identifiable in any reports, articles, presentations or other outcomes arising from the research.

### Ancillary and post-trial care

Following completion of the study, parents/guardians will be provided with individualised feedback about their child’s participation and recommendations for post-trial care if deemed appropriate/necessary (e.g. ongoing group or individual therapy). We do not anticipate any significant harms resulting from participation in the intervention nor believe that there will be any need for compensation for loss or damages.

### Dissemination of findings

Findings will be communicated to parent/guardians in brief written reports and with parental consent, reports will also be sent to relevant healthcare professionals (e.g. neurologists, general practitioners [GPs]) involved in the child’s care. Findings will be disseminated in peer-reviewed publications and conference presentations, as well as forums run through The University of Sydney and the Australian Research Council Centre of Excellence for Cognition and its Disorders, with whom the project is affiliated. Investigators involved in designing/implementing the intervention or those who make significant contributions to the final write up of results will be eligible for authorship. Professional writers will not be used.

## Discussion

The primary purpose of this paper was to outline the protocol for a pilot study that will assess feasibility, acceptability and potential efficacy of a novel cognitive behavioural intervention with ToM training for children with epilepsy. To our knowledge, this is the first psychosocial treatment to be developed and trialled to address social competence problems in children with epilepsy. The intervention is critically needed as social difficulties are common and pervasive in children with epilepsy, yet evidence-based interventions to treat these problems do not currently exist.

By tailoring the intervention to address specific impairments in ToM and related cognitive skills that are common among children with epilepsy, we believe that there is greater possibility of children improving in both ToM and social competence. The intervention directly targets impairments in advanced cognitive and affective ToM that have been found to relate to social competence problems in children with epilepsy, aged 8 to 12 years old [5–7]. It includes strategies to assist with cognitive skills that develop alongside ToM (i.e. language, EF), which are commonly impaired in young epilepsy patients [25, 28, 29], as well as CBT exercises to promote generalisation of ToM to broader social skills. In addition, various aspects of treatment delivery have been included to optimise the learning environment for children (e.g. same age peers, small groups, parental involvement).

Individual benefits for children who participate in the study may include an improvement in ToM, communication and social skills. Parents may benefit from learning about how to best assist their child with the ToM and social difficulties that they face. Findings obtained in standardised neuropsychological assessments will provide parents with additional information about the child’s general cognitive skills (IQ, language, EF), which may be beneficial for addressing children’s functioning at home and school. Nevertheless, as is the case with any clinical trial, there are no guaranteed benefits for participants and this will be discussed with parents prior to enrolment and clearly outlined in PICFs. On a broader scale, the study has the potential to enhance the knowledge base and skillset of clinicians who routinely work with children with epilepsy, which is likely to improve detection and provision of appropriate psychosocial support.

Limitations of the current study include small sample size and omission of a control group. This will preclude us from performing statistical analyses (i.e. power calculations) on secondary outcome measures (i.e. ToM, social competence) that would allow us to estimate the sample size needed for a larger scale trial and will not allow us to assess the feasibility and pragmatics of randomisation. Nevertheless, given the preliminary nature of the intervention, we believe that the case series design is the most ethical and feasible approach. As we only plan to recruit children with TLE and GGE (8 to 12 years old), we will not be able to determine suitability of the intervention for children with other forms of epilepsy or adolescents with epilepsy (> 12 years old). Given that adolescents are at different stages of social development, we did not think that administering the same intervention to a single group would be appropriate or efficacious. In future work, the intervention may be extended to adolescents and other clinical groups.

## Conclusion

The proposed intervention represents a new area of cognitive remediation for epilepsy patients and addresses a major gap in clinical care of this patient group. Results from the pilot study will be used to refine and modify research methods and decide whether the intervention should be evaluated in a larger scale trial. If successful, the study would provide preliminary support for the first evidence-based intervention to address social impairments in children with epilepsy, which has the potential to improve longer term social outcomes for this group.

## Additional files

Additional file 1: SPIRIT 2013 Checklist: Recommended items to address in a clinical trial protocol and related documents. Completed SPIRIT checklist. (DOC 119 kb)

Additional file 2: Template for Intervention Description and Replication (TIDieR) checklist. (DOCX 33 kb)

Additional file 3: Participant information sheet and consent forms (PICFs) for the trial. PICFs that have been approved by the governing ethics committee for the trial. Parent and child PICFs and the withdrawal of consent form are provided within the single document. (DOCX 1296 kb)

## Abbreviations

ANZCTR: Australia and New Zealand Clinical Trials Register
ASD: Autism spectrum disorder
AWMA: Automated Working Memory Assessment
BTPS: Barriers to Treatment Participation Scale
CBCL: Child Behavior Checklist
D-KEFS: Delis-Kaplan Executive Functions System
EF: Executive functioning
FSIQ: Full Scale Intellectual Quotient
GASE: Global Assessment of Severity of Epilepsy
GGE: Genetic generalised epilepsy
GP: General practitioner
HREC: Human Research Ethics Committee
MRC: Medical Research Council
PICF: Participant information and consent forms
PPVT-4: Peabody Picture Vocabulary Test–Fourth Edition
RCT: Randomised controlled trial
SDQ: Strength and Difficulties Questionnaire
SPIRIT: Standard Protocol Items: Recommendations for Interventional Trials
SPP: Self-perception profile
TEI: Treatment Evaluation Inventory
TIDieR: Template for Intervention Description and Replication
TLE: Temporal lobe epilepsy
ToM: Theory of Mind
TOMI: Theory of Mind Inventory
WASI-II: Wechsler Abbreviated Scale of Intelligence for Children–Second edition
