# Supplementary material for: A novel cognitive behavioural intervention with Theory of Mind (ToM) training for children with epilepsy: protocol for a case series feasibility study
Source: Pilot Feasibility Stud. 2019 Jan 19;5:12. doi: 10.1186/s40814-019-0393-x (PMC6339364; doi:10.1186/s40814-019-0393-x)
Supplement: Supplementary file 3 — Participant information sheet and consent forms (PICFs) for the trial. PICFs that have been approved by the governing ethics committee for the trial. Parent and child PICFs and the withdrawal of consent form are provided within the single document. (DOCX 1296 kb) [file 40814_2019_393_MOESM3_ESM.docx]

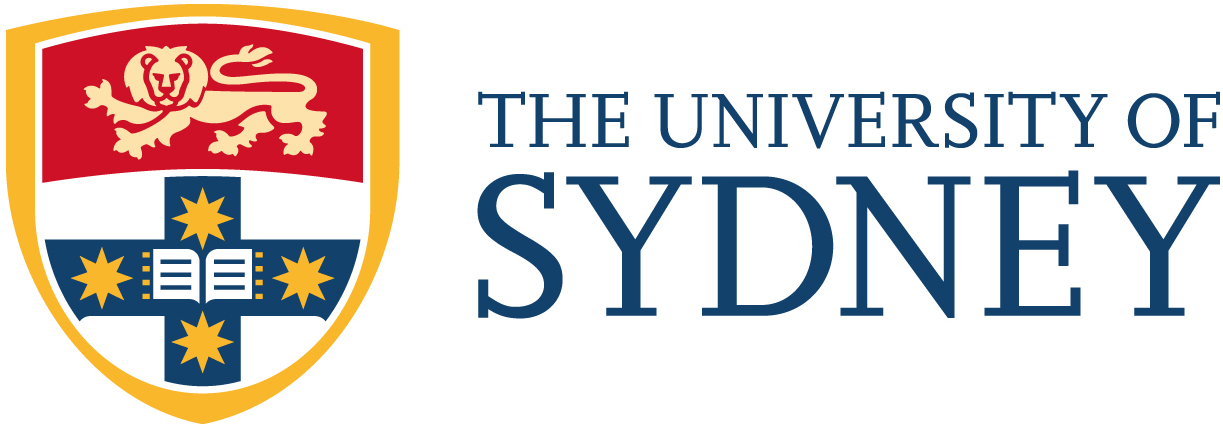

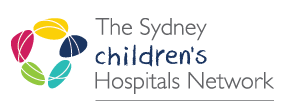


**Participant Information Sheet/Consent Form – Parent/Guardian**

**Interventional Study** - *Parent/Guardian consenting on behalf of participant*

Sydney Children’s Hospital

| **Title** | Cognitive behavioural intervention for social perspective taking in children with epilepsy: a feasibility study |
| --- | --- |
| **Short Title** | Cognitive behavioural intervention child epilepsy |
| **Protocol Number** | HREC/18/SCHN/21 |
| **Coordinating Principal Investigator/ Principal Investigator** | Associate Professor Suncica Lah |
| **Associate Investigator(s)** | Miss Elizabeth Stewart  Dr Deepak Gill  Dr Richard Webster  Dr John Lawson  Dr Anna Mandalis  Associate Professor Cathy Catroppa |
| **Location** | University of Sydney  Children’s Hospital at Westmead  Children’s Hospital at Randwick |

**Part 1 What does your child’s participation involve?**

**1 Introduction**

This is an invitation for your child to take part in this research project, which is testing a new therapeutic intervention for children with epilepsy. The new intervention is designed to improve children’s social perspective taking skills and overall social competence.

This Participant Information Sheet/Consent Form tells you about the research project. It explains the assessments and intervention sessions involved in the study. Knowing what is involved will help you decide if you want your child to take part. Please read this information carefully. Ask questions about anything that you don’t understand or want to know more about. Participation in this research is voluntary. If you do not wish your child to take part, they do not have to. Your child will receive the best possible care whether or not they take part.

If you decide you want your child to take part in the research project, you will be asked to sign the consent section. By signing it you are telling us that you:

• Understand what you have read

• Consent to your child taking part in the research project

• Consent for your child completing the assessments and workshops that are described in this sheet

**2 What is the purpose of this research?**

This study aims to assess a new therapeutic intervention for children with epilepsy. The intervention is designed to target social perspective taking skills. This is a pilot/feasibility study, which means we are interested in whether the intervention is engaging and beneficial for your child, whether activities can be completed in the allocated time frame, and whether it runs smoothly as an overall program. We also want to identify any barriers that you may encounter in attending sessions and completing the program. We will answer these questions with information you and your child provide to us throughout the program and by asking independent, experienced clinicians to evaluate the program.

**3 What is the intervention?**

The intervention is designed to improve social and perspective taking skills in children with epilepsy. It is a new intervention that has been developed specifically for children with epilepsy. The intervention has been designed based on findings from clinical research showing that children with epilepsy have many more social difficulties than their peers and are more likely to be bullied and socially isolated. Children with epilepsy also have difficulty solving social conflicts and often struggle to interpret and respond to social cues. Despite this, no interventions exist to target these social difficulties.

This intervention aims to improve children’s ability to: (1) communicate their thoughts and feelings to social partners (2) perspective take about the thoughts and feelings of other people, which is important for socialising and empathetic responding, and (3) respond appropriately in social situations.

**3 What does participation in this research involve?**

| **A summary of the main participation requirements of the study**   1. Workshops. Your child will be asked to attend 4 small-group workshops (2 to 3 hours each), held over 4 consecutive weeks, at the Psychology Clinic at the University of Sydney. These will be conducted outside of school hours. Parents/guardians will attend a brief review session at the end of each day with your child. 2. Assessments. Your child will complete 4 one-to-one assessments (15 to 35 minutes each) to examine his/her social and perspective taking skills. These will be conducted at your home or at the Psychology Clinic on a day that your child is attending a workshop. Parents/guardians will be asked to complete questionnaires while your child completes these assessments. 3. Weekly ratings. You and your child will be asked to answer 3 to 6 multiple-choice questions each week via telephone about his/her social participation during the week. |
| --- |

**Enrolment, eligibility and consent**

If you decide to participate in this study, we will telephone you to ask some brief screening questions and check your child is eligible for the study. During this phone call, we will arrange the first assessment session (at the clinic or your home) and provide you with the dates of workshops. Prior to the initial assessment, we will ask you to sign and return the consent form.

**Workshops**

The workshops will be held in 2 to 3 hour sessions, over 4 consecutive weeks at the Psychology Clinic at the University of Sydney. During the workshops, your child will participate in activities with 1 to 5 other children who are a similar age. These sessions involve interactive tasks including videos, cartoons, stories, role-plays, worksheets and group discussions. The videos included are from popular children’s cartoon films and are G or PG rated. Please inform the researcher if you would like to view these in advance. All activities are designed to be engaging and enjoyable for children and teenagers and are similar to activities that would be used in a typical child therapy setting. Children will be given a workbook at the start of the program, which we ask you to bring to each session.

While your child is attending the workshops, we ask parents to remain in the waiting room, as we find that this is least distracting and results in the best participation from children. You can leave the facility while your child is attending the workshops and return prior to the end of their scheduled session. If you choose to do this, we will ask you to provide us with a telephone number that we can use to contact you. Parents/guardians will be asked to attend a brief (30 minute) review session at the end of each training day with your child. Altogether, the workshops should last 2 to 3 hours each, including the parent review session and breaks.

**Assessment sessions**

Your child will complete 4 assessments (15 to 35 minutes) with a researcher, in which they will complete story tasks that assess social and perspective taking. These assessments will be conducted at your home or at the Clinic on a workshop day. Therefore, you will not have to make any additional visits to the Clinic. Assessments will be conducted 4 weeks before the workshop, on the first day of the workshop, on the last day of the workshop and 4 weeks after the workshops have finished. The timing and location of these assessments will be arranged with you in advance.

Parents/guardians will be asked to complete questionnaires at these same 4 points in time. These will be sent via an email link and can be completed online. We ask that you complete these within 24 hours of your child’s assessment to ensure that the information gathered is accurate and consistent. These questionnaires gather information about your child’s social and emotional functioning and perspective-taking skills, which will help us determine whether the training program is effective.

**Weekly ratings**

Throughout the study, you and your child will be asked to complete 3 to 6 multiple-choice questions (1 to 5 minutes) each week about your child’s social participation during the week. These will be answered online and we will provide a reminder email to help you remember. The reason we are collecting these weekly measures is to gain an understanding of how your child is progressing over the course of the study, whether there are improvements that are maintained or whether additional difficulties emerge that need to be addressed.

**Feedback and debriefing**

After the final workshop, we will arrange a one-on-one debriefing session with you. The debriefing session will give you the opportunity to ask questions and provide feedback about the training. We will ask you some questions about how useful and achievable you found the program to be. This meeting can be conducted in person or over the telephone, whichever is more convenient for you. We will also provide a brief report summarising your child’s participation and containing recommendations for follow-up with appropriate services, if this is required. The report will be sent to you within 6 weeks of the follow-up assessment. It will also be sent to your child’s treating neurologist, and with your consent, to any other clinicians involved in your child’s care, such as your family doctor. A summary of the study timeline is provided in Table 1.

Table 1. Study timeline of intervention and assessment procedures for children

| Week | 1 | 2 | 3 | 4 | 5 | 6 | 7 | 8 | 9 | 10 | 11 | 12 |
| --- | --- | --- | --- | --- | --- | --- | --- | --- | --- | --- | --- | --- |
| Location | Home or Clinic |  |  |  | Clinic | Clinic | Clinic | Clinic |  |  |  | Home or Clinic |
| Workshops |  |  |  |  | X  2 – 3 hours | X  2 – 3 hours | X  2 – 3 hours | X  2 – 3 hours |  |  |  |  |
| Assessments | X  30 min |  |  |  | X  10 min |  |  | X  10 min |  |  |  | X  20 min |
| Weekly ratings | X  1–5 min | X  1–5 min | X  1–5 min | X  1–5 min | X  1–5 min | X  1–5 min | X  1–5 min | X  1–5 min | X  1–5 min | X  1–5 min | X  1–5 min | X  1–5 min |
| Total time commitment each week | 35 min | 1–5 min | 1–5 min | 1–5 min | 2–3.25 hours | 2 – 3 hours | 2 – 3 hours | 2–3.25 hours | 1–5 min | 1–5 min | 1–5 min | 25 min |

**4 Other relevant information about the research project**

There are no costs associated with participation in this research project. All assessments and workshops will be provided to you free of charge. The study is an open-label pilot/feasibility trial with no control group. This means that all children who enrol in this study will complete the same intervention, which is described above. There is no chance that your child will be assigned to a waitlist or control group. The study is a collaboration between researchers and clinicians at the University of Sydney, The Children’s Hospital at Westmead and the Sydney Children’s Hospital at Randwick.

**6 Does my child have to take part in this research project?**

Participation in any research project is voluntary. If you do not wish for your child to take part, they do not have to. If you decide that they can take part and later change your mind, you are free to withdraw your child from the project at any stage.

If you do decide that your child can take part, you will be given this Participant Information and Consent Form to sign and you will be given a copy to keep.

Your decision for your child to take part or not, or to take part and then be withdrawn, will not affect their routine treatment, relationship with those treating them, or their relationship with the University of Sydney, The Children’s Hospital at Westmead or the Sydney Children’s Hospital at Randwick.

**7 What are the alternatives to participation?**

Alternate options to participation in this study include seeking out private treatment with a registered psychologist who has experience working therapeutically with children and adolescents with epilepsy. The study researchers will discuss these options with you before you decide whether or not your child can take part in this research project. You can also discuss the options with your child’s local doctor.

**8 What are the possible benefits of taking part?**

We cannot guarantee or promise that your child will receive any benefits from this research. However, possible benefits may include improvements to your child’s social, communication, and perspective taking skills. They may also show improvements in their understanding of non-literal actions and language, such as understanding irony, jokes, metaphors and figures of speech. We expect to see overall improvements in children’s self-awareness and ability to communicate their thoughts and feelings, as well as an understanding of how to modify their social responses based on the situation and social partner. Participation in group workshops with other children may be beneficial in enhancing your child’s confidence in socialising with peers.

**9 What are the possible risks and disadvantages of taking part?**

We do not anticipate any risks from taking part in this study. All activities included are safe and appropriate for children and teenagers. Some tasks will be harder than others, but it is not expected that any of the tasks will be too difficult. Children will be provided with guidance and feedback during the workshops. They will be reminded that there is no right or wrong answer and encouraged to give activities their best try.

The researcher(s) conducting assessments and workshops are registered psychologist(s) who have experience working with children and adolescents and will be able to respond sensitively and take appropriate steps should you or your child experience distress. If you or your child become upset or distressed as a result of participation in the research, the researchers will be able to arrange for counselling or other appropriate support. Any counselling or support will be provided free of charge.

**10 Can my child have other treatments during this research project?**

Whilst your child is participating in this research project, they should continue to take any usual medication they have been prescribed for their epilepsy or other health conditions. It is important to tell the researchers about any changes in medications during the research project.

If your child is receiving concurrent treatment with a psychologist or counsellor, it is important to discuss this with the researchers prior to the study. Concurrent therapies could interfere with the intervention and make it difficult to determine whether improvements are due to the intervention or another treatment your child is receiving. The researchers will discuss options with you before commencement of the study.

**11 What if I withdraw my child from this research project?**

If you decide to withdraw your child from the project, please notify a member of the research team before you withdraw them. This notice will allow the research team to discuss this withdrawal with you. If you do withdraw the participant during the research project, the researchers will not collect additional personal information, although personal information already collected will be retained to ensure that the results of the research project can be measured properly and to comply with law.

**12 Could this research project be stopped unexpectedly?**

It is unlikely that the research project will be stopped unexpectedly. The study will continue to run even if only one child is attending sessions. Therefore, your child will receive the full training program regardless of whether other children are able to complete the program or not. In the unlikely event that the study cannot continue, you will be notified by the researchers and provided with debriefing and referral to appropriate services, if required.

**13 What happens when the research project ends?**

Once you have completed the follow-up assessment, nothing further will be required of you or your child. If we think your child would benefit from further interventions, we will discuss this with you and provide referral to appropriate services. We can provide you with a summary of study outcomes once they are finalised, which may take up to 12 months after the follow-up assessment has been completed. If you wish to receive information about the overall study outcomes please inform the researchers.

**Part 2 How is the research project being conducted?**

**14 What will happen to information about my child?**

By signing the consent form you consent to the research staff collecting and using personal information about your child for the research project. Any information obtained in connection with this research project will remain confidential and will only be used for the purpose of this research project. It will be disclosed only with your permission, or as required by law.

All information collected will be scanned and stored in password protected files on a computer database at the University of Sydney and retained for a minimum of 15 years, after which time it will be securely deleted. Your child’s information will be assigned a unique code and no identifying information will be retained in the database.

It is anticipated that the results of this research project will be published and presented in a variety of forums. In any publication and/or presentation, information will be provided in such a way that your child cannot be identified.

Information about your child’s participation in this research project may be recorded in their health records. For instance, the final report that is sent to you will also be sent to your child’s treating neurologist. We will also send this to other clinicians involved in your child’s care, with your consent.

In accordance with relevant Australian and New South Wales privacy and other relevant laws, you have the right to request access to the participant’s information collected and stored by the study team. You also have the right to request that any information with which you disagree be corrected. Please contact the study team member named at the end of this document if you would like to access the participant’s information.

**15 Who is organising and funding the research?**

This is a student project that will contribute towards to Doctor of Philosophy (PhD) of Miss Elizabeth Stewart. Elizabeth is a registered psychologist, who holds general registration with the Psychology Board of Australia (PSY0001782791) and has experience working in a clinical and research capacity with children with epilepsy. Elizabeth will be supervised by Associate Professor Suncica Lah, who will meet regularly with Elizabeth and oversee the project. It is a self-funded project, meaning no external funding has been provided. If you wish to discuss the project either during or after the study, you are welcome to contact either Elizabeth or A/Prof Lah. Contact details are provided in Section 17..

**16 Who has reviewed the research project?**

All research in Australia involving humans is reviewed by an independent group of people called a Human Research Ethics Committee (HREC). The ethical aspects of this research project have been approved by the HREC of the Sydney Children’s Hospital Network (SCHN). This project will be carried out according to the National Statement on Ethical Conduct in Human Research (2007). This statement has been developed to protect the interests of people who agree to participate in human research studies.

**17 Further information and who to contact**

If you would like any further information concerning this project and wish to contact the study researchers, please contact Elizabeth Stewart (ph: 0404 060 549; email: [este7735@uni.sydney.edu.au](mailto:este7735@uni.sydney.edu.au)) or Associate Professor Suncica Lah (ph: 02 9351 2641, email: [suncica.lah@sydney.edu.au](mailto:suncica.lah@sydney.edu.au)).

**This project has been approved by Sydney Children’s Hospitals Network Human Research Ethics Committee. If you have any concerns about the conduct of this study, please do not hesitate to contact the Executive Officer of the Ethics Committee (02 9845 3066) or via email SCHNethics@health.nsw.gov.au and quote approval number HREC/18/SCHN/21).**

**Expression of Interest Form – Parent/Guardian**

Please complete and return this Expression of Interest form in the supplied reply-paid envelope, or email Elizabeth Stewart at este7735@uni.sydney.edu.au to indicate whether or not you would like to participate. The Researcher from the University of Sydney will contact you via telephone if they do not receive a response from you within two weeks of mailing this letter to confirm your interest or not.

✂----------------------------------------------------------------------------------------------------------------------

I would like to be contacted regarding the intervention study on social perspective taking in children with epilepsy:

Yes  No

My child does / does not have a diagnosis of epilepsy.

Signed: _____________________________________________________________

Name: _____________________________________________________________

Date: _____________________________________________________________

My preferred contact details are:

Home Telephone Number: ________________________________

Business Telephone Number: __________________________

Email Address: ________________________________

My preferred contact times are:

_________________ AM / PM to _________________ AM / PM

*OR*

_________________ AM / PM to _________________ AM / PM

*OR*

_________________ AM / PM to _________________ AM / PM

**Consent Form – Parent/Guardian**

| **Title** | Cognitive behavioural intervention for social perspective taking in children with epilepsy: a feasibility study |
| --- | --- |
| **Short Title** | Cognitive behavioural intervention child epilepsy |
| **Protocol Number** | HREC/18/SCHN/21 |
| **Coordinating Principal Investigator/**  **Principal Investigator** | Associate Professor Suncica Lah |
| **Associate Investigator(s)** | Miss Elizabeth Stewart  Dr Deepak Gill  Dr Richard Webster  Dr John Lawson  Dr Anna Mandalis  Associate Professor Cathy Catroppa |
| **Location** | University of Sydney  Children’s Hospital at Westmead  Children’s Hospital at Randwick |

**Declaration by Parent/Guardian**

I have read the Participant Information Sheet or someone has read it to me in a language that I understand.

I understand the purposes, procedures and risks of the research described in the project.

I give permission for my child’s doctors, other health professionals, hospitals or laboratories outside this hospital to release information to The University of Sydney concerning my child’s epilepsy and treatment for the purposes of this project. I understand that such information will remain confidential.

I have had an opportunity to ask questions and I am satisfied with the answers I have received.

I freely agree to my child participating in this research project as described and understand that I am free to withdraw them at any time during the research project without affecting their future health care.

I understand that I will be given a signed copy of this document to keep.

|  |  | |  | | | |  |
| --- | --- | --- | --- | --- | --- | --- | --- |
|  | Name of Child (please print) |  | | | | |  |
|  |  |  | | | | |  |
|  | Signature of Child | |  | | Date |  |  |
|  |  | |  | |  |  |  |
|  | Name of Parent/Guardian (please print) | | |  | | |  |
|  |  | | |  | | |  |
|  | Signature of Parent/Guardian | |  | | Date |  |  |
|  | | | | | | | |

**Declaration by Study Researcher^†^**

I have given a verbal explanation of the research project, its procedures and risks and I believe that the parent/guardian has understood that explanation.

|  | | | | | | | |
| --- | --- | --- | --- | --- | --- | --- | --- |
|  | Name of Study Doctor/  Senior Researcher^†^ (please print) | |  | | |  | |
|  | | | | | |  | |
|  | Signature |  | | Date |  | |  |
|  | | | | | | | |

^†^ A senior member of the research team must provide the explanation of, and information concerning, the research

**Form for Withdrawal of Participation – Parent/Guardian**

| **Title** | Cognitive behavioural intervention for social perspective taking skills in children with epilepsy |
| --- | --- |
| **Short Title** | Cognitive behavioural intervention child epilepsy |
| **Protocol Number** | HREC/18/SCHN/21 |
| **Coordinating Principal Investigator/**  **Principal Investigator** | Associate Professor Suncica Lah |
| **Associate Investigator(s)** | Miss Elizabeth Stewart  Dr Deepak Gill  Dr Richard Webster  Dr John Lawson  Dr Anna Mandalis  Associate Professor Cathy Catroppa |
| **Location** | University of Sydney  Children’s Hospital at Westmead  Children’s Hospital at Randwick |

**Declaration by Parent/Guardian**

I wish to withdraw the child from participation in the above research project and understand that such withdrawal will not affect their routine treatment, relationships with those treating them or the relationship with the University of Sydney, the Children’s Hospital at Westmead or the Children’s Hospital at Randwick.

|  |  | |  | | | |  |
| --- | --- | --- | --- | --- | --- | --- | --- |
|  | Name of Child (please print) |  | | | | |  |
|  |  |  | | | | |  |
|  | Signature of Child | |  | | Date |  |  |
|  |  | |  | |  |  |  |
|  | Name of Parent/Guardian (please print) | | |  | | |  |
|  |  | | |  | | |  |
|  | Signature of Parent/Guardian | |  | | Date |  |  |
|  | | | | | | | |

In the event that the parent/guardian’s decision to withdraw is communicated verbally, the Study Doctor/Senior Researcher will need to provide a description of the circumstances below.

|  |
| --- |

**Declaration by Study Researcher^†^**

I have given a verbal explanation of the implications of withdrawal from the research project and I believe that the parent/guardian has understood that explanation.

|  | | | | | | |
| --- | --- | --- | --- | --- | --- | --- |
|  | Name of Study  Researcher^†^ (please print) | |  | | |  |
|  | | | | | |  |
|  | Signature |  | | Date |  |  |
|  | | | | | | |

^†^ A senior member of the research team must provide the explanation of, and information concerning, withdrawal from the research project.

Note: All parties signing the consent section must date their own signature


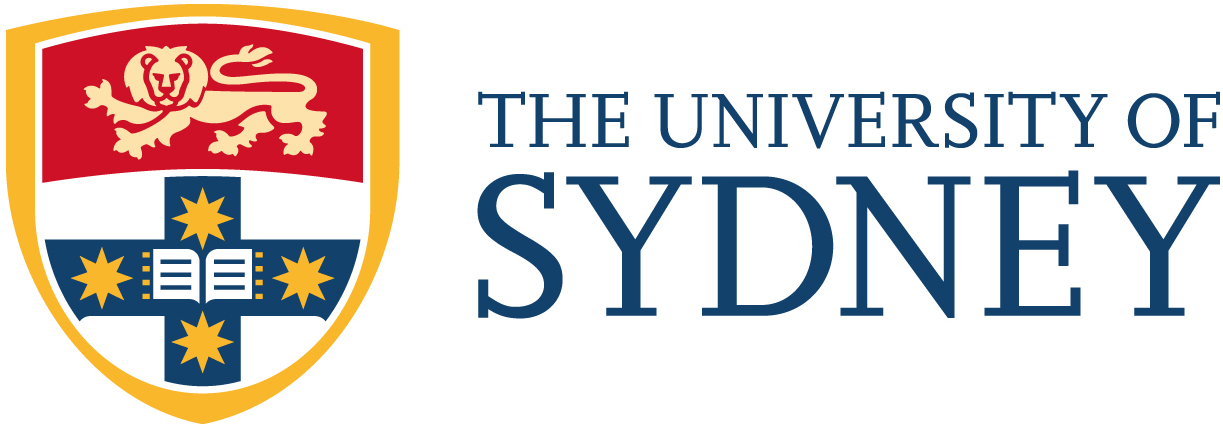

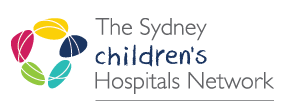


**Participant Information Sheet/Consent Form – Child**

**Interventional Study** – *Information for children; Parent/Guardian consenting on behalf of participant*

Sydney Children’s Hospital

| **Title** | Cognitive behavioural intervention for social perspective taking in children with epilepsy: a feasibility study |
| --- | --- |
| **Short Title** | Cognitive behavioural intervention child epilepsy |
| **Protocol Number** | HREC/18/SCHN/21 |
| **Coordinating Principal Investigator/ Principal Investigator** | Associate Professor Suncica Lah |
| **Associate Investigator(s)** | Miss Elizabeth Stewart  Dr Deepak Gill  Dr Richard Webster  Dr John Lawson  Dr Anna Mandalis  Associate Professor Cathy Catroppa |
| **Location** | University of Sydney  Children’s Hospital at Westmead  Children’s Hospital at Randwick |

This is a booklet that has been put together to help you decide if you would like to take part in our research study. The study is testing whether a new program, which teaches social and perspective taking skills, helps you. The reason we are doing the study is because we know that lots of children and adolescents, including those with epilepsy, have difficulties with social and perspective taking skills, and this can make socialising hard. At the moment, there are no programs to help children and adolescents with epilepsy develop these skills.

**1 Who is doing the study?**

This study is being run by researchers at the University of Sydney, the Children’s Hospital at Westmead, and the Sydney Children’s Hospital at Randwick. All the people who are doing the study work with children with epilepsy, such as deciding what medicines you should take, understanding how you learn best at school, and helping with feelings and friendships.

**2 What is the study about?**

We are trying to find out whether a new program will help you understand other people’s thoughts and feelings and know how to respond in social situations. Another way of saying this is to help you learn perspective taking and social skills. We want to know whether you find the activities in the program fun and enjoyable, and whether you use the skills you learn at home and school. We also want to know what things you don’t like about the program. Finding out these things will help us improve the program so that it can help other children like you.

**3 What will I have to do if I choose to take part?**

If you decide you want to take part, there will be a number of activities and tasks that you will complete. These are explained below.

First, a researcher will visit your home or you will come to the Psychology Clinic to complete some tasks for around 15 to 35 minutes. The tasks involve answering questions about characters in stories, such as why someone said something or why they acted in a particular way.

About 6 weeks after you complete these first tasks, you will visit the Psychology Clinic at the University of Sydney. Here, you will get to do activities in a workshop with 1 to 5 other children who are about the same age as you. The activities will include watching videos and cartoons, reading stories, doing role-plays, and doing some other group activities. This will go for about 2 to 3 hours in total. You will be given lots of breaks so you don’t get tired. The activities will help you learn about perspective taking, which is really important for making and keeping friends. While you are doing these activities, your parent/guardian will stay in the waiting room and come in for the last 30 minutes so that we can explain to them what you have learned that day. One week later, you will come back for another workshop with the same children. You will do this once per week, for 4 weeks.

About 6 weeks after the last workshop, we will visit your home again, or you will visit the Psychology Clinic, to complete the same tasks that you did when you first met the researcher. This time, it will only take 15 minutes. After you have completed these tasks, we will arrange a meeting with you and your parent/guardian so that you can tell us whether you found the program fun, enjoyable and helpful.

Finally, for each week that you are in the program (about 12 weeks), you will receive a phone call or email from the researcher and be asked to answer 3 to 6 multiple-choice questions about how you felt about your friendships and social participation that week. The questions take about 1 to 5 minutes, and you will answer the same questions each week. Your parent/guardian will help you answer these questions.

**4 Do I have to take part in this research project?**

No you don’t. If you say no, that is ok. It is up to you. Even if you take part at the beginning and change your mind later on and don’t want to be a part of the study, that is okay as well. All you need to do is tell the researcher that you don’t want to take part anymore. You also don’t need to answer any question that you don’t want to, that is okay too.

**5** **Will anyone know that I am taking part or hear about what I tell you?**

No, no-one will know what information you gave to the researchers. You can tell them whatever you want and no-one will know that it came from you. The only time the researchers would have to tell someone is if anyone hurt you or upset you in any way. The researchers would also have to tell someone if you said you might hurt yourself or someone else.

**6 Is there anything that might make me upset if I take part in the research?**

We do not think that any of the activities or tasks in this study will upset you. They are safe and appropriate for children and teenagers that are your age. Some tasks will be harder than others, but none of the tasks should be too difficult. The researchers will be there to help you with the activities.

If anything you talk about during the research does make you upset you can stop the research. Your parents/carers will be told and you will be given the names of people you can talk to about what is making you upset, if that is what you want to do. The researcher can help you do that.

**7 What will happen to the information I tell you?**

All information about you will remain confidential. This means that only you and the researchers will know what you have told us. We plan to write reports in scientific journals on what we find in this study. These reports will show how a group of children and adolescents responded to the program. Your name will not be written in any of these reports, so nobody will know that you participated. Once the study has been completed the information will be stored in a locked room on a password protected computer at the University of Sydney and will be destroyed after 15 years.

**8 Further information and who to contact**

It is your choice whether you participate or not. If you change your mind during the study and want to stop participating, that is okay too. You should tell your parent/guardian and they will discuss this with the study researchers.

If you have any questions about the research project or you want to talk about it, please contact usElizabeth Stewart (ph: 0404 060 549; email: [este7735@uni.sydney.edu.au](mailto:este7735@uni.sydney.edu.au)) or Associate Professor Suncica Lah (ph: 02 9351 2641, email: suncica.lah@sydney.edu.au).

**This project has been approved by Sydney Children’s Hospitals Network Human Research Ethics Committee. If you have any concerns about the conduct of this study, please do not hesitate to contact the Executive Officer of the Ethics Committee (02 9845 3066) or via email SCHNethics@health.nsw.gov.au and quote approval number HREC/18/SCHN/21)**
